# Insight into the Effects of Sous Vide on Cathepsin B and L Activities, Protein Degradation and the Ultrastructure of Beef

**DOI:** 10.3390/foods9101441

**Published:** 2020-10-12

**Authors:** Yantao Yin, Jailson Pereira, Lei Zhou, Jose M. Lorenzo, Xiaona Tian, Wangang Zhang

**Affiliations:** 1 Key Laboratory of Meat Processing and Quality Control, Ministry of Education China, Jiangsu Collaborative Innovation Center of Meat Production and Processing, Quality and Safety Control, College of Food Science and Technology, Nanjing Agricultural University, Nanjing 210095, China; yantaoyin@126.com (Y.Y.); jailsemedo@hotmail.com (J.P.); judgeaie@163.com (L.Z.); 2017108069@njau.edu.cn (X.T.); 2 Centro Tecnológico de la Carne de Galicia, rúa Galicia n° 4, Parque Tecnológico de Galicia, San Cibrao das Viñas, 32900 Ourense, Spain; jmlorenzo@ceteca.net; 3 Área de Tecnología de los Alimentos, Facultad de Ciencias de Ourense, Universidad de Vigo, 32004 Ourense, Spain

**Keywords:** sous vide, tenderness, cathepsins, protein proteolysis

## Abstract

This study aimed to evaluate the effects of sous vide cooking (SV) on beef tenderness and its underlying potential mechanism. Beef *semimembranosus* (SM) were subjected to SV treatments at 45 °C, 55 °C and 65 °C for 4 h. Compared with control samples (CK, cooked at 75 °C until a core temperature of 72 °C was attained), SV treatment significantly promoted the release of cathepsin B and cathepsin L from lysosomes and decreased the shear force of beef SM (*p* < 0.05). In comparison with CK, samples treated with SV had more hydrolysis of myosin heavy chain and obtained higher myofibrillar fragmentation index, collagen solubility as well as longer sarcomere length (*p* < 0.05). The current study showed that the proteolysis of myofibrillar protein and collagen induced by cathepsin B and cathepsin L, and the limited longitudinal shrinkage together contributed to the improvement of beef tenderness upon SV.

## 1. Introduction

Sous vide cooking (SV) cooking has become increasingly popular in centralized kitchens, catering operations and restaurants. SV is a cooking method in which foods are vacuum-packaged and cooked at a controlled temperature. A typical SV for beef processing is performed at 50–70 °C for 2–24 h depending on the intramuscular connective tissue, myofibrillar protein components, thickness and type of the meat [1]. Compared with the traditional high-temperature cooking, SV could maintain higher mineral content of meat and minimize lipid oxidation [2]. Furthermore, SV is widely used to process ready-to-eat products for modern consumers who prefer the convenience of foods [3].

Tenderness is an important factor influencing consumer choices and satisfactions with meat. Although several studies reported that SV contributed to the improvement of meat tenderness [2,4], the underlying mechanism about how SV improve beef tenderness remains poorly understood. The improvement of meat tenderness with cooking is related to the weakening of both myofibrillar proteins and collagen [5]. The endogenous calpains and cathepsins are considered as major proteolytic systems responsible for the protein degradation during post-mortem aging and processing, thus contributing to the textural changes [6,7]. The activity of cathepsin B + L in bovine cooking loss exudate was still detected after cooking at 55 °C for 7.5 h [8]. In consideration of the relatively high thermal stability of cathepsin B (Cth B) and cathepsin L (Cth L), it is reasonable to speculate that Cth B and Cth L may participate in the improvement of beef tenderness with SV treatment. To test this hypothesis, changes in activities of Cth B and Cth L, collagen solubility, ultrastructure of collagen observed with scanning electron microscope (SEM), hydrolysis of myofibrillar protein as well as ultrastructure of beef *semitendinosus* during SV treatment were investigated in this study.

## 2. Materials and Methods

### 2.1. Sample Preparation

Four Luxi yellow cattle (250 ± 15 kg) were slaughtered at Shandong Hongan (Group) Co. Ltd. (Binzhou, China) according to the “Operating Procedures of Cattle Slaughter” of the National Standards of P. R. China (GB12694-2006). In order to reduce the impact of environmental factors on the consistency of beef samples during postmortem, the semimembranosus (SM) muscles from both carcass sides were removed at 24 h postmortem as described in previous studies [2,9]. Subsequently, the connective tissue and subcutaneous fat were removed, and then each muscle was cut into 60 × 50 × 30 mm along with the myofibrillar direction. Next, the cuts were randomly divided into four groups. The SV samples were vacuum packaged in polyethylene pouches using a vacuum packaging machine (DC-860S, Promarksvac, San Francisco, CA, USA). The packaged samples were immersed in a water bath (20 L) and cooked with sous vide circulator (Polyscience, Niles, IL, USA) at 45 °C, 55 °C and 65 °C for 4 h. Each treatment was designated as SV45, SV55 and SV65, respectively. As the control (CK), sample was placed in a cooking bag and cooked under 78 °C until a core temperature of 72 °C was attained. Once the cooking period was over, samples were immediately submerged into ice water (0 °C) for 30 min. The cooking loss exudates of samples treated by SV45, SV55 and SV65 as well as exudate from raw SM were collected for measuring Cth B and Cth L activities.

### 2.2. Cth B and Cth L Activity

Cth B and Cth L activities in exudate were performed according to the method of Christensen et al. [8] with slight modifications. For Cth B and Cth L activities in exudate, 15 μL of RW exudate or cooking loss exudate of SV samples was mixed with 135 μL reaction buffers (340 mM sodium acetate, 60 mM acetic acid, 4 mM EDTA, 0.1% Brij 35, pH 5.0). Then, the mixture was incubated at 40 °C for 10 min with 100 μL synthetic fluorogenic substrate (12.5 μM in methanol). The Z-Arg-Arg-AMC and the Z-Phe-Arg-AMC (Sigma, St. Louis, MO, USA) were corresponding to cathepsin B and B + L fluorogenic substrate, respectively. The fluorescence values were measured with excitation and emission wavelengths of 355 nm and 460 nm using a microplate reader (M2, Molecular Devices, Sunnyvale, CA, USA). The unit of enzyme activity (U) was defined as the amount of AMC released per minute (μmol) at 40 °C. Cth L activity was calculated with cathepsin L + B − Cth B. A background control was performed using buffer instead of exudate.

For Cth B and Cth L activities in muscle, beef sample (0.5 g) was homogenized for 2 × 15 s at 12,000 rpm using a homogenizer (T10, IKA, Staufen, Germany) in 2.5 mL buffer (50 mM sodium citrate, 1 mM EDTA, 0.2% Triton X-100, pH 5.0). Then, the extract was centrifuged at 4 °C (20 min, 12,000× *g*). The supernatant was collected for the measurement of enzyme activity as described above.

### 2.3. Collagen Solubility

Meat samples (1 g) or exudate (2 mL) was hydrolyzed with 5 mL HCl (6 M) for 16 h at 110 °C. The hydroxyproline content in the hydrolysate was determined by the method of Latorre et al. [10]. The content of collagen was counted as the hydroxyproline content was multiplied by 7.25. The solubility of collagen was calculated according to the following formula:

(1) $$\mathrm{Collagen}\;\mathrm{solubility}\;=\;\frac{\;\mathrm{soluble}\;\mathrm{collagen}}{\mathrm{soluble}\;\mathrm{collagen}+\;\mathrm{insoluble}\;\mathrm{collagen}}\times 100\%$$

where soluble collagen: collagen content in the exudate and insoluble collagen: collagen content in the meat residue.

### 2.4. Preparation of Intramuscular Connective Tissue

Intramuscular connective tissue was prepared as the method of Aktas et al. [11]. Each sample (10 g) mixed with 100 mL of precooled buffer (0.1 M KCl + 0.02 M KH_2_PO_4_/K_2_HPO_4_, pH 6.0) was homogenized at 8000 rpm for 2 × 20 s. The homogenate and the connective tissue adhered to the blade were filtered through a 25-mesh sieve. The filter residue was resuspended in the precooled buffer and homogenized at 8000 rpm for 15 s. This procedure was repeated three times. After the third filtration, the filter residue was washed by stirring in 100 mL of distilled water for 30 s. Then, the flushing was centrifuged at 2000× *g* for 10 min and the precipitate was obtained.

### 2.5. Differential Scanning Calorimetry (DSC)

The endothermal transition of intramuscular connective tissue was investigated according to Aktas et al. [11] using DSC (Q20, TA Instruments, New Castle, DE, USA). Intramuscular connective tissue samples (15–17 mg) were accurately weighed into aluminum pans and hermetically sealed. Samples were thermally scanned from 20 to 90 °C at 10 °C/min. As reference, an empty pan was heated in the same way. The peak maximum temperature (Tm, °C) was analyzed by the software (Version 4.5A, TA Instruments).

### 2.6. Scanning Electron Microscope (SEM)

The ultrastructure was observed using a SEM (SU-8010; Hitachi, Tokyo, Japan). Samples (6 × 6 × 0.2 mm) were cut from the central parts of treated beef pieces, and fixed with 2.5% glutaraldehyde in 0.1 M PBS (pH 7.4) for 36 h. Then, samples were washed with 0.1 M PBS (pH 7.4) for three times, following dehydrated with gradient ethanol solutions (30%, 50%, 70%, 90% and 100%). Immediately, the samples were washed three times using *tert*-butyl alcohol (Sigma-Aldrich, Shanghai, China) and frozen at −20 °C. After freeze dried and gold-coated, the images were observed under SEM.

### 2.7. Preparation of Myofibrillar Protein

Myofibrillar protein was prepared as previously described by Kang et al. [12]. Beef samples (5 g) with 25 mL of precooled extraction buffer A (0.1 M NaCl, 2 mM MgCl_2_, 10 mM K_2_HPO_4_, 1 mM EGTA, pH 7.0) were homogenized at 12,000 rpm for 2 × 30 s. The homogenate was centrifuged at 2000× *g* (10 min, 4 °C), and then the pellet was washed twice with 25 mL of extraction buffer. Continuously, the pellet was washed three times with 25 mL extraction buffer B (0.1 M NaCl, pH 6.0). Finally, the recovered pellet was considered as myofibrillar protein and suspended in suspension buffer (0.6 M NaCl, 20 mM phosphate, pH 6.0). The protein concentration was measured using BCA Protein Assay Kit (Thermo Scientific, Waltham, MA, USA).

### 2.8. Sodium Dodecyl Sulfate Polyacrylamide Gel Electrophoresis (SDS-PAGE)

SDS-PAGE was performed based on the report of Fu et al. [6]. Briefly, myofibrillar protein (2 mg/mL) was mixed with equal volume of SDS-PAGE loading buffer, and the mixture was boiled at 95 °C for 5 min. Next, 8 μL mixture or 5 μL molecular standard marker (Thermo Fisher, Shanghai, China) was loaded on each lane of precast gel (GenScript, 10% polyacrylamide, 10 wells). The electrophoresis was performed using a Mini-Protean Tetra System (Bio-Rad Laboratories, Hercules, CA, USA) at 70 V for 30 min, and then at 110 V for about 70 min at 4 °C. After separation, the gel was stained with Coomassie Brilliant Blue (0.1%) for 40 min, and then the gel was decolorized with decolorizing solution (10% methanol, 10% acetic acid; 1:1) for several times until the bands became clear.

### 2.9. Myofibril Fragmentation Index (MFI)

MFI was measured following the procedure as described by Wang et al. [13]. Beef samples (2 g) were homogenized at 15,000 rpm (2 × 30 s) in 40 mL of MFI buffer (100 mM KCl, 1 mM EDTA, 20 mM phosphate buffer, pH 7.0). Then the homogenate was centrifuged at 1000× *g* (4 °C, 15 min). After centrifugation, the precipitate was suspended in MFI buffer and centrifuged again. Next, the precipitation was resuspended in 20 mL of MFI buffer. The protein concentration of the final suspension was adjusted to 0.5 mg/mL using MFI buffer. The absorbance of the suspension was measured at 540 nm using a spectrophotometer. MFI was calculated as the absorbance of the solution was multiplied by 200.

### 2.10. Ultrastructure and Sarcomere Length

The ultrastructure and the sarcomere length were observed as described by the procedures of Kang et al. [12]. Briefly, samples of 3 × 3 × 5 mm were taken along with the direction of myofibril and fixed in 2.5% glutaraldehyde (0.1 M PBS pH 7.4). Then, samples were cut into 1.5 × 2 × 2 mm pieces and washed with PBS buffer (0.1 M pH 7.4) for 3 times and 10 min each time. Subsequently, the smaller blocks were post-fixed in 1% osmium tetroxide for 3 h at 25 °C. Samples were then dehydrated with a graded series of ethanol (50, 70, 90 and 100%), infiltrated and polymerized in in epoxy resin (Durcupan; Sigma-Aldrich). Ultrathin sections (70 nm thickness) were cut and collected on copper grids. After stained using 2% uranyl acetate (in 50% ethanol) and lead citrate, respectively, the sections were observed under TEM (H-7650, Hitachi Corporation, Tokyo, Japan). Five TEM images were randomly obtained from each sample and lengths of 10 sarcomeres from each image were evaluated by Image-Pro Plus (5.1, Media Cybernetics, Bethesda, MD, USA). The sarcomere length was expressed as the average of 50 measurements.

### 2.11. Shear Force

Shear force was assessed according to the method described by Fu et al. [6]. The cooled meat sample was cut into 3 × 1 × 1 cm blocks, and three blocks of the same sample were used for the shear force measurements. The shear force was measured using a muscle tenderness instrument (C-LM, Northeast Agricultural University, Haerbin, China).

### 2.12. Statistical Analysis

Five replicates of all samples were analyzed unless specified. Data analysis was performed using SPSS 25.0 (SPSS Inc., Chicago, IL, USA). The difference between each treatment was calculated using one-way analysis of variance (ANOVA) and the means separated by Turkey’s multiple comparison test. The significant difference was set at *p* < 0.05. The result was expressed as mean ± standard error.

## 3. Results and Discussion

### 3.1. Cth B and Cth L Activities in Exudate

**Figure 1:**
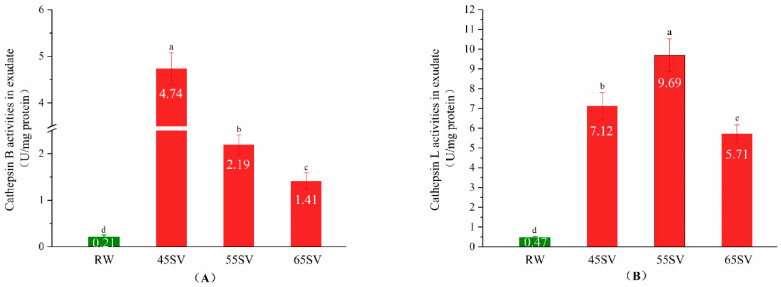
Cathepsin B (**A**), Cathepsin L (**B**) activity in exudate of RW and SV with different temperature. Different letter (a–d) statistically indicates the significant differences on the activity of cathepsin B (*p* < 0.05, *n* = 5).

Cth B and Cth L are located in lysosomes, and their tenderizing effects on meat depend on whether they could be released from lysosomes [8]. The present study evaluated the release of Cth B and Cth L by measuring their activities in the cooking loss exudates, and the results are shown in Figure 1. As shown in Figure 1A, the Cth B activity in the raw (RW) sample exudate was only 0.21 U/mg protein. Interestingly, SV treatment significantly increased the Cth B activity in cooking loss exudate by 21.57-fold, 9.43-fold and 5.71-fold for SV45, SV55 and SV65 (*p* < 0.05), respectively. A similar phenomenon was observed for cathepsin L (Figure 1B). It is well known that Cth B and Cth L are water-soluble. The pronounced increment of Cth B and Cth L activities in SV samples exudate suggested that they were released from lysosomes, and then migrated into cooking loss exudate as stated by Christensen et al. [8]. There is limited information available in the literature regarding the release of the cathepsins from lysosome with cooked meat products. Whereas, the release of cathepsins from lysosomes could be regulated by external factors, such as lactic acid [14], and high-pressure [15]. Previous investigators suggested that cooking destroyed the antioxidant system of muscles and facilitated the formation of reactive oxygen species (ROS) [16]. Lysosome is a classical target of ROS, and it is generally accepted ROS could initiate lipid peroxidation of lysosome membrane, which in turn induces membrane damage [17,18]. Therefore, it can be speculated that the release of Cth B and Cth L with SV treatment may be related to the damage of lysosomes membrane.

### 3.2. Cth B and Cth L Activities in Muscle

**Figure 2:**
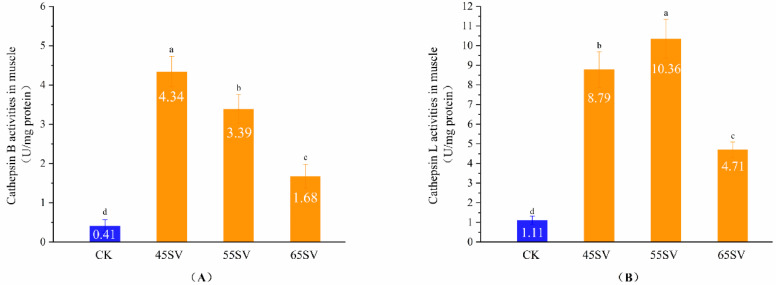
Cathepsin B (**A**), Cathepsin L (**B**) activity in muscle of CK and SV with different temperature. Different letter (a–d) statistically indicates the significant differences on the activity of cathepsin B (*p* < 0.05, *n* = 5).

The results of Figure 1 indicate that Cth B and Cth L were released from lysosomes. However, the hydrolyses of beef proteins are mainly influenced by cathepsins in muscle. Therefore, we further evaluated Cth B and Cth L activities in muscle, and the results are shown in Figure 2. Samples in SV groups had significantly higher activities of Cth B and Cth L compared to that of CK (*p* < 0.05). This phenomenon might be associated with two different aspects. First, the longer cooking time in SV gave more time for the release of Cth B and Cth L from lysosomes compared with CK. Second, Cth B and Cth L exist as zymogens, and they are sensitive to temperature [19]. Previous studies indicated that when the heating temperature surpassed 65 °C, the activities of Cth B and Cth L in duck decreased quickly [20]. Therefore, the highest cooking temperature in CK (72 °C) might be responsible for its low activities of Cth B and Cth L. To some extent, our results are in agreement with recent study, which reported Cth B and Cth L in beef brisket were more heat stable with 50 °C SV than that of 70 °C SV [21]. Also, previous study reported the activity of Cth B and Cth L in pork was higher at 55 °C than 70 °C [22].

Cth B showed the maximal activity at SV45 among the three SV treatments (Figure 2A). Although Cth B activity decreased with the increasing temperature, the SV65 sample still maintained 43% of the activity of Cth B. Different from Cth B, Cth L had the highest activity with samples treated at 55 °C (Figure 2B). The difference in Cth B and Cth L activities treated with different SV temperature might be related to their variations of optimum temperature.

### 3.3. Change in Collagen

#### 3.3.1. DSC

**Figure 3:**
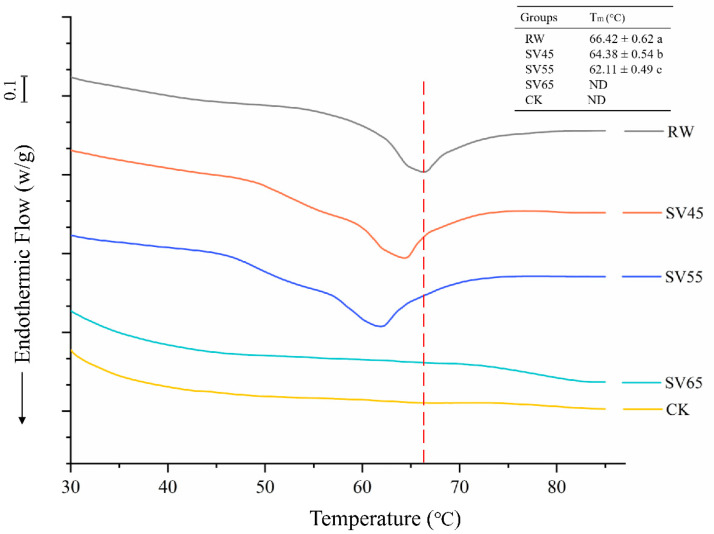
DSC endotherm of collagen from RW, CK and SV sample. Different letter (a–c) in the same column indicates statistically significant differences (*p* < 0.05, *n* = 5).

Collagen is the main component of connective tissue in muscle. Collagen consists of a triple helix structure maintained by a high content of covalent linkages and hydrogen bonds which enable it to resist thermal dissolution [23]. DSC is a powerful tool to evaluate the kinetics of thermal denaturation and structural stability of collagen. As shown in Figure 3, RW samples exhibited a single endothermic transition peak with a temperature maximum (Tm) at 66.42 °C. The Tm of RW samples was similar to previously reported, which corresponded to the denaturation temperature of collagen [24]. Compared to RW, the Tm of SV45 and SV55 treated samples moved to lower temperature, and there was a significant decrease (*p* < 0.05) in thermal denaturation temperature with the Tm of 64.38 and 62.11 °C, respectively. For the SV65 and CK samples, the endothermic peak disappeared.

The endothermic peak corresponds to the denaturation of collagen from the triple helix structure. Bertola et al. [25] reported that the endothermic peak of collagen disappeared after heating at 66 °C for 5 min or 68 °C for 0 min. Therefore, the disappearances of endothermic peak observed in SV65 and CK samples might be due to their higher cooking temperature. The significant reduction of Tm collagen in SV45 and SV55 samples indicated the remarkably decreased stability of their highly ordered structures. It is generally believed that heat-induced kinetic energy can disrupt the hydrogen bond, van der Waals force and electrostatic attraction between collagen fibers, thus resulting in collagen solubilization [26]. On the other hand, Cth B and Cth L could degrade proteoglycan in collagen or directly hydrolysis collagen [27]. It is therefore reasonable to infer that the effect of long cooking time cooperated with Cth B and Cth L might be responsible for the reduced T_m_ occurred in SV45 and SV 55 samples.

#### 3.3.2. Collagen Solubility

The solubility of collagen is usually positively correlated with the tenderness of cooked meat [23]. Compared with CK, the collagen solubility of samples treated with SV45, SV55 and SV65 increased by 12%, 27% and 12%, respectively (Table 1). The improvement of collagen solubility by SV could be due to the accumulated more thermal energy by longer cooking time (4 h), which destroyed the collagen fibers. Li et al. [28] suggested that prolonging cooking time under low temperature conditions (53 °C or 58 °C) promoted the dissolution of collagen in pork. However, Latorre et al. [10] reported that there was no significant change in collagen solubility with cooking from 0–25 h at 60 °C. The variations in the collagen solubility observed in different studies could be related to animal species, sex and age. Interestingly, it was observed that samples treated by SV55 obtained higher collagen solubility than that of SV65 under the same SV time. This phenomenon suggested that the improvement of collagen solubility might be due to the synergies of SV temperature and Cth B and Cth L. Supporting this view, Beltran et al. [27] reported that Cth B and Cth L could hydrolyze collagen, thus promoting the collagen solubility. Similarly, Berge et al. [29] reported Cth B + L increased the collagen solubility with injecting lactic acid during beef post mortem.

#### 3.3.3. SEM

**Figure 4:**
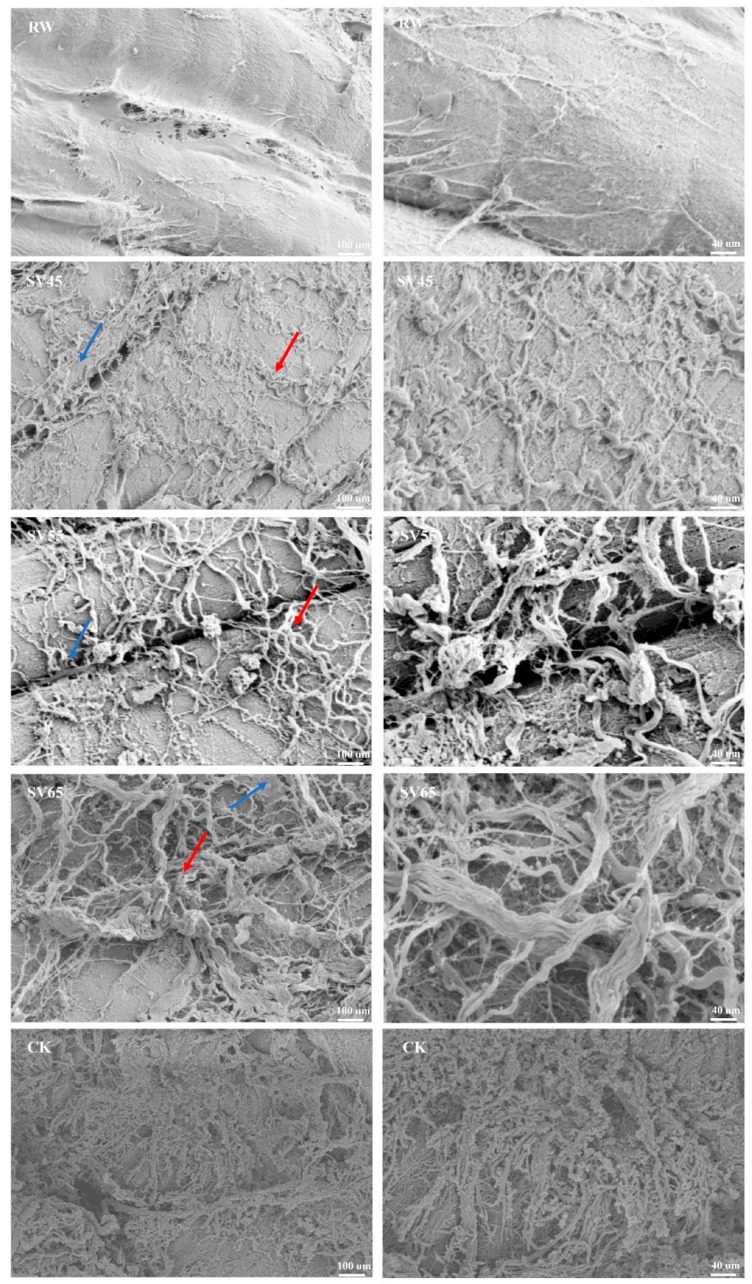
SEM images of intramuscular connective tissues from RW, SV sample and CK.

In order to further observe the effect of SV on collagen, the ultrastructure of intramuscular connective tissues was observed with SEM. The improvement of the cooking beef tenderness is related to the changes in intramuscular connective tissues of perimysium and endomysium [23]. Endomysium surrounds each individual muscle fiber, and perimysium is a continuous network that integrates all the fascicles. As shown in Figure 4, it is difficult to observe endomysium in RW sample, which might be due to the closely connection of perimysium and endomysium as well as overlapping of vision due to longitudinal cut. For CK sample, the endomysium was also hardly observed. In contrast, the endomysium (marked with blue arrow) and perimysium (marked with red arrow) could be distinguished in SV sample. In addition, intramuscular connective tissues were smoothly wrapped on the myofibrils in RW sample, which looked like a dense membrane. Whereas, it was observed a dissolution and fracture in the SV45 sample and less intramuscular connective tissues at SV55 sample compared to other treatment groups. Coarse filamentous aggregations were observed in the SV65 sample and there were numbers of granular aggregates in CK sample. The thin and loose intramuscular connective tissues observed in SV sample might be related to the loss of soluble collagen [28]. The changes in ultrastructure of intramuscular connective tissues were consistent with the results of collagen solubility (Table 1). These evidences highlighted the synergistic effect of SV temperature and Cth B and Cth L on collagen solubility.

### 3.4. SDS-PAGE and MFI

**Figure 5:**
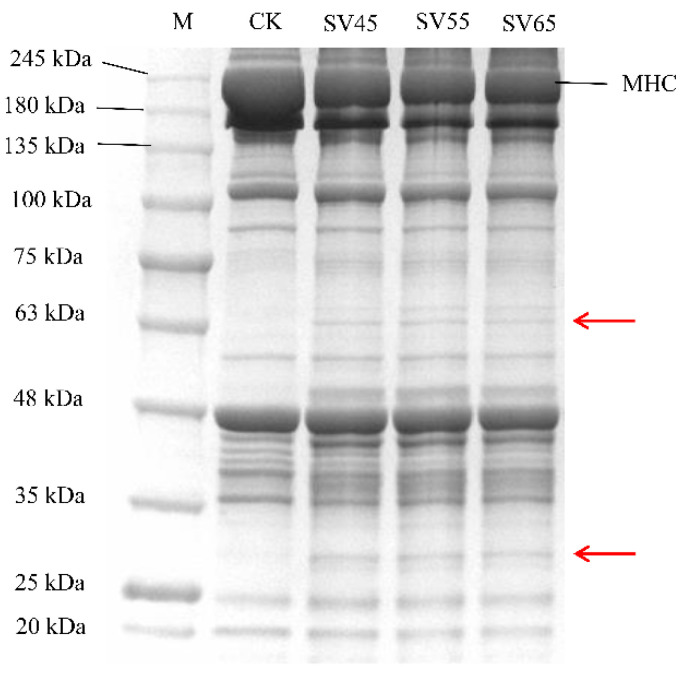
SDS-PAGE of myofibrillar proteins extracted from beef semitendinosus of CK and SV samples. M: molecular weight markers, MHC: myosin heavy chain.

Myofibrillar protein accounts for 55% of beef proteins and its degradation greatly contributes to the tenderness improvement of the meat. The myofibrillar protein profiles from samples treated with SV and CK are presented in Figure 5. Compared with CK, the intensity of myosin heavy chain (MHC) band in SV lanes decreased. Furthermore, new bands with small molecular weight appeared in SV lanes (as shown in the red arrows), indicating that SV resulted in the hydrolysis of MHC. MHC is the main component of myosin, which constitutes approximately 55–60% of the myofibrillar fractions in skeletal muscle [30].

Many studies demonstrated that Cth B and Cth L are involved in the hydrolysis of MHC. For example, Ertbjerg et al. [14] observed more degradation of MHC with the existence of higher activity of Cth B and Cth L in the lactic acid marinated beef. In the present study, the activities of Cth B and Cth L in SV samples were significantly higher compared to that in CK (Figure 2). Thus, it can be speculated that the degradation of MHC in SV samples could be associated with the activation of Cth B and Cth LTo further assess the effect of SV on myofibrillar protein proteolysis, MFI was determined. As shown in Table 1, SV significantly increased the MFI compared with CK (*p* < 0.05). MFI is an indication of the integrity of myofibrils, and the increase of MFI is associated with the degradation of MP during post-mortem and processing [12,13]. The increase of MFI in SV samples further confirmed that SV treatment promoted myofibrillar protein proteolysis.

### 3.5. Ultrastructure of Myofibre and Sarcomere Length

**Figure 6:**
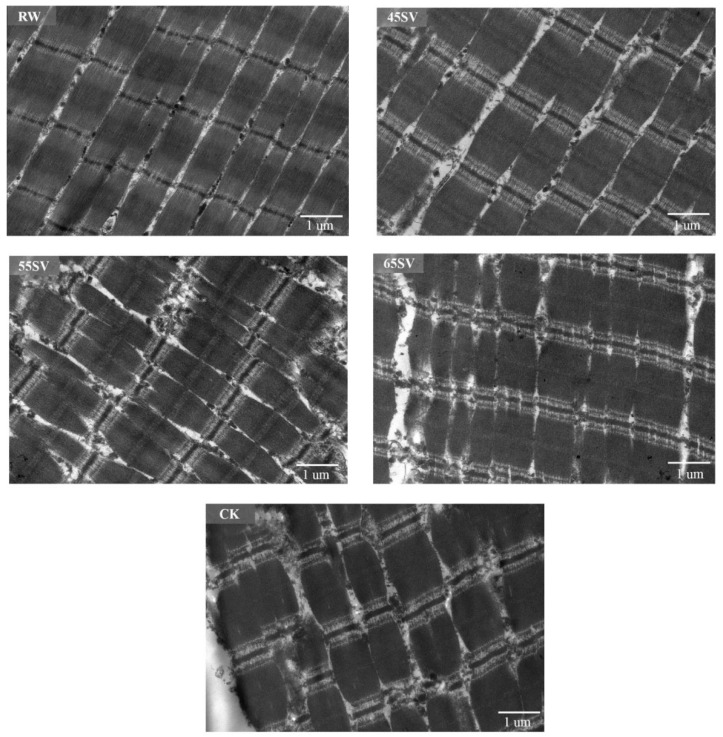
TEM images of beef *semitendinosus* from RW, CK and SV sample.

**Figure 7:**
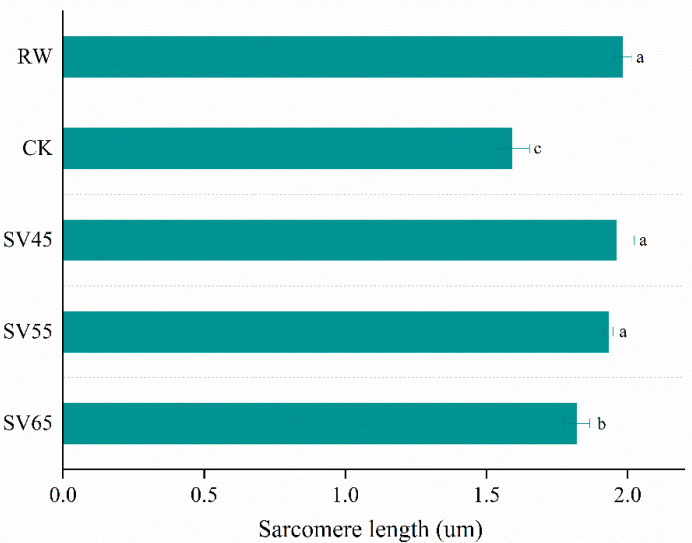
Sarcomere length (B) of beef *semitendinosus* from RW, CK and SV sample. ^a–c^ Different letter indicates statistically significant differences (*p* < 0.05, *n* = 50).

To give a visualized proof of changes in myofibril and sarcomere length, the ultrastructure was observed using TEM. As shown in Figure 6, the sarcomere in RW sample was arranged neatly with clear I-band, A-band, and Z-line, which are the typical features of the myofibrillar ultrastructure [12]. Compared to RW, some fragmentations and gaps were observed between Z line and I band junctions in both CK and SV samples (Figure 6). The morphological changes of ultrastructure could be related to the denaturation of myofibrillar proteins [31]. To further analyze the effect of SV on sarcomere, the sarcomere length was measured in this study. As shown in Figure 7, CK and SV65 significantly reduced the sarcomere length compared to RW (*p* < 0.05). It is worth noting that there were no significant differences in sarcomere length among RW, SV45 and SV55 samples as shown in Figure 7 (*p* > 0.05). Previous research suggests denaturation of collagen could result shrinkage of sarcomere along longitudinal axis [32]. Therefore, the contraction of the sarcomere in CK and SV65 samples could be explained by their higher cooking temperatures. In contrast, relatively mild cooking temperatures of SV45 and SV55 treatments had little effect on sarcomere length. In addition, the proteolysis destroyed the integrity of sarcomere, which might also contribute to less sarcomere contraction in SV samples, as explained by Wang et al. [33].

### 3.6. Shear Force

The shear force values from CK and different SV treatments are shown in Table 1. CK samples had a shear force of 62.34 N/cm^2^, which is consistent with previous report from Hou et al. [34]. Compared with CK, SV significantly reduced the shear force of beef SM, and the lowest shear force was obtained with SV55 treatment (*p* < 0.05). These results indicate that SV is an effective way to improve beef tenderness. Our result is consistent with the study of Bhat et al. [2]. These authors reported that SV (60 °C for 4 h) significantly improved the meat tenderness of dairy cows. Based on the results above, a hypothesis about the improvement of beef tenderness by SV has been proposed. SV could destroy the membrane of lysosomes, which led Cth B and Cth L to release into sarcoplasm and myofibrils. Meanwhile, the mild cooking temperature and longer cooking time provided a suitable condition for Cth B and Cth L to degrade myofibrillar protein and collagen, thus improving the tenderness of beef by SV. In addition, as most studies showed, there was a significant negative correlation between sarcomere length and meat tenderness [32]. Therefore, the limited longitudinal shrinkage as well as the more proteolysis of myofibrillar protein and collagen could together contribute to the improvement of beef tenderness by SV.

## 4. Conclusions

The current study reveals that SV is effective in improving the tenderness of beef SM. It was observed that SV could promote the release of Cth B and Cth L from lysosomes by comparing the activities of Cth B and Cth L in the exudate of RW and SV samples. Besides, SV maintained higher Cth B and Cth L activities in muscle than CK. Moreover, comparison to CK, SV significantly promoted the dissolution of collagen and the degradation of myofibrillar protein as evidenced by the increased collagen solubility, MFI and hydrolysis of MHC. In addition, SV remarkably avoided sarcomere shortening compared to CK. Therefore, the proteolysis of myofibrillar protein and collagen induced by Cth B and Cth L coupled with the limited longitudinal shrinkage in myofibril might contribute to the improvement of beef tenderness by SV. Additional studies with proteomics are required to focus on the changes of proteins and cathepsins in SV beef, which could provide a comprehensive understanding of the improvement in the tenderness upon SV methods.

## Figures and Tables

**Table 1:** Effect of SV treatment on collagen solubility, MFI and shear force.

	CK	SV45	SV55	SV65
Collagen solubility (%)	3.54 ± 0.11 ^c^	3.74 ± 0.17 ^c^	4.49 ± 0.19 ^a^	3.96 ± 0.10 ^b^
MFI	35.37 ± 1.66 ^c^	44.83 ± 1.67 ^a^	47.66 ± 2.42 ^a^	39.67 ± 2.47 ^b^
Shear force (N/cm^2^)	62.34 ± 2.50 ^a^	55.28 ± 1.31 ^b^	46.69 ± 1.47 ^c^	52.84 ± 1.61 ^b^

^a–c^ Different letters in the same row indicate statistically significant differences (*p* < 0.05, *n* = 5).
